# Metabolomics-Based Study of the Protective Effect of 4-Hydroxybenzyl Alcohol on Ischemic Astrocytes

**DOI:** 10.3390/ijms25189907

**Published:** 2024-09-13

**Authors:** Tian Xiao, Xingzhi Yu, Jie Tao, Liping Yang, Xiaohua Duan

**Affiliations:** Yunnan Key Laboratory of Dai and Yi Medicines, Yunnan University of Chinese Medicine, Kunming 650500, China; xiaot7107@163.com (T.X.); 18313150021@163.com (X.Y.); 19188572767@163.com (J.T.); ynylp81@163.com (L.Y.)

**Keywords:** 4-hydroxybenzyl alcohol, ischemic stroke, astrocytes, blood–brain barrier, mitochondria

## Abstract

Ischemic stroke is a common and dangerous disease in clinical practice. Astrocytes (ASs) are essential for maintaining the metabolic balance of the affected regions during the disease process. 4-Hydroxybenzyl alcohol (4HBA) from *Gastrodia elata* Bl. has potential neuroprotective properties due to its ability to cross the blood–brain barrier. In an in vitro experiment, we replicated the oxygen–glucose deprivation/reoxygenation model, and used methyl thiazoly tertrazolium, flow cytometry, kits, and other technical means to clarify the protective effect of 4HBA on primary ASs. In in vivo experiments, the 2VO model was replicated, and immunofluorescence and immunohistochemistry techniques were used to clarify the protective effect of 4HBA on ASs and the maintenance of the blood-brain barrier. Differential metabolites and related pathways were screened and verified using metabolomics analysis and western blot. 4HBA noticeably amplified AS cell survival, reduced mitochondrial dysfunction, and mitigated oxidative stress. It demonstrated a protective effect on ASs in both environments and was instrumental in stabilizing the blood–brain barrier. Metabolomic data indicated that 4HBA regulated nucleic acid and glutathione metabolism, influencing purines, pyrimidines, and amino acids, and it activated the N-methyl-D-aspartate/p-cAMP-response element binding protein/brain-derived neurotrophic factor signaling pathway via N-methyl-D-aspartate R1/N-methyl-D-aspartate 2C receptors. Our findings suggest that 4HBA is a potent neuroprotective agent against ischemic stroke, enhancing AS cell survival and function while stabilizing the blood–brain barrier. The N-methyl-D-aspartate/p-cAMP-response element binding protein/brain-derived neurotrophic factor signaling pathway is activated by 4HBA.

## 1. Introduction

Ischemic stroke, a common and dangerous disease in clinical practice, has become a common medical problem that threatens human health due to its high incidence and disability rate, and it has also shown a trend of increasingly affecting younger people [1]. A thromboembolism in the brain deprives the surrounding brain tissues of blood oxygen and energy, thus triggering a series of pathological reactions and causing the death of a large number of brain cells [2]. Although the initial thrombolytic treatment can instantly restore the blood flow supply, brain cell damage in the ischemic core area will be further aggravated, and the infarcted area will be further enlarged, which is called cerebral ischemia/reperfusion injury (CIRI) [3]. However, a large number of brain cells in the ischemic semi-dark zone around the ischemic core can still be salvaged [4]. Therefore, we should seek more therapeutic means to ameliorate brain cell damage and reduce CIRI, with the aim of developing more ideal therapies.

Brain cells are mainly composed of neurons and glial cells, with the total volume of astrocytes (ASs) accounting for about 50% of the total brain volume [5]. They are connected to each other through powerful gap junctions, forming an extended cellular network with long-distance communication capabilities [6]. As the only “glycogen storehouse” in the brain, ASs provide nutritional support to the surrounding neuronal cells through complex signaling to maintain neuronal functioning [7]. In an anaerobic environment, the death of ASs was amplified when they were cocultured with neurons, suggesting that they may accelerate their own cell death by providing energy to damaged neurons [8]. ASs were more resistant to ischemia than neurons when cultured alone [9]. However, ASs are the first to be attacked during oxygen deprivation due to their synaptic terminals being connected to capillaries, and thus, absorbing nutrients [10]. Therefore, ASs may be an important target to reduce neuronal damage and ameliorate CIRI.

ASs occupy an important position in regulating the metabolism of substances in ischemic regions, and metabolomics is the most powerful tool to reflect the overall metabolic status of cells [11]. Metabolomics is the comprehensive study of the small-molecule metabolites present in a biological system. It provides the metabolic status of an organism at a specific time and can be used to understand pathological processes [12]. It can also screen specific differentially abundant metabolites in each group to reveal their underlying metabolic networks [13]. Metabolic pathways are highly identical among species, and the mechanisms and functions found in biological models are susceptible to the assessment of human metabolomics [14]. DL-3-N-Butylphthalide was extracted from the seeds of *Apium graveolens* Linn; it plays an anti-cerebral ischemia role by regulating the metabolism of molecules including ATP and antioxidant factors in the brain [15]. A study has found that artemisinin has the effect of inhibiting the pyroptosis pathway, and thus, fighting CIRI through serum metabolomics [16]. The above studies aimed to reveal not only the mechanism of drug action but also the differentially abundant metabolites that can be used as markers for disease diagnosis, which can provide a reference for the precise use of drugs in the treatment of CIRI.

**Figure 1:**
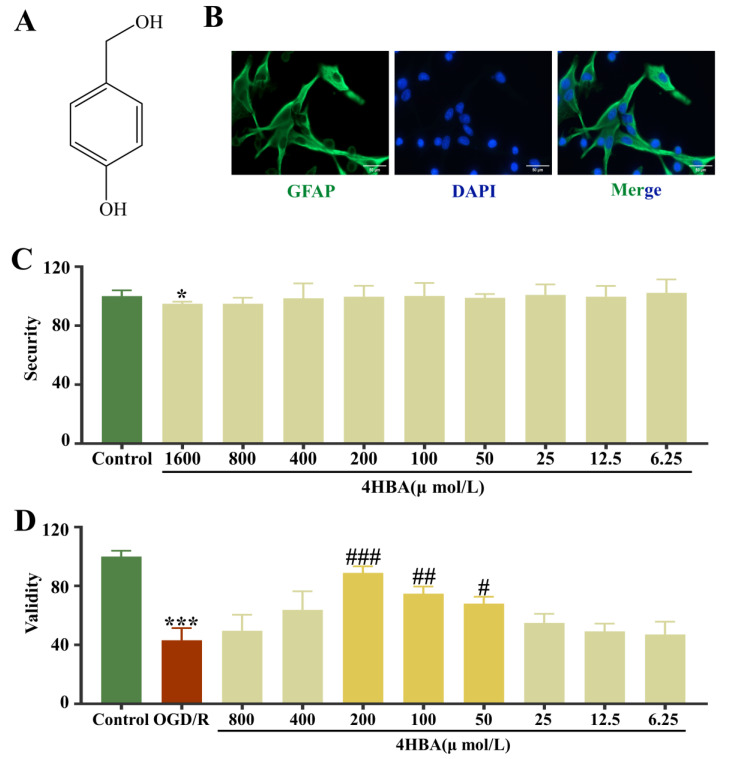
Chemical structure of 4HBA (**A**). Immunofluorescence staining plots of GFAP (bar = 50 μm) (**B**). Effect of 4HBA at different concentrations on the viability of normal ASs (**C**). Effect of 4HBA on the viability of OGD/R ASs (**D**). Data are presented as the mean ± standard deviation (SD). ### *p* < 0.001, ## *p* < 0.01, # *p* < 0.05 vs. the control group. *** *p* < 0.001, * *p* < 0.05 vs. the OGD/R group. Abbreviations: OGD/R, oxygen–glucose deprivation/reoxygenation; 4HBA, 4-hydroxybenzyl alcohol.

Modern research has found that *Gastrodia elata* Bl. contains rich phenolic compounds which dilate blood vessels; it improves hypoxia tolerance and other pharmacological activities, and it is the main active ingredient in the treatment of cardiovascular and cerebrovascular diseases [17]. The results of our team’s previous research suggest that 4-hydroxybenzyl alcohol (4HBA) has the strongest active role among several phenolic ingredients of *Gastrodia elata* Bl. in the protection of neuronal cells against CIRI [18]. 4HBA has a variety of pharmacological effects such as sedation–hypnosis, alleviating cognitive dysfunction, antidepressant activity, fighting neuroinflammation, alleviating oxidative stress, and reducing acute nephritis [19,20,21,22,23]. At the same time, it has a strong transmembrane capacity, so it is easily absorbed in the intestinal tract and is more likely to cross the blood–brain barrier (BBB) to reach the focal point of the disease [24]. In an in vitro model of Zn^2+^-induced CIRI, 4HBA was found to have a strong protective effect on ASs and neurons damaged by Zn^2+^ toxicity [25]. Notably, unlike 3-hydroxybenzyl alcohol and 2-hydroxybenzyl alcohol, 4HBA specifically induced protein disulfide isomerase expression, which resulted in a significant amelioration of CIRI [26] (Figure 1A). Thus, 4HBA has significant potential in protecting ASs and may become an effective neuroprotective agent at the brain cellular and molecular levels. However, the intervention effect of 4HBA on astrocytes in CIRI and its related mechanisms are still unclear.

Therefore, in the present study, we replicated the oxygen–glucose deprivation/reoxygenation (OGD/R) in vitro model and the 2VO rat in vivo model while utilizing liquid chromatography–tandem mass spectrometry (LC-MS/MS) metabolomics analysis techniques. We want to clarify the protective effect of the active ingredient 4HBA on the repair of ASs and the fight against CIRI under the intervention of *Gastrodia elata* Bl., and to reveal its potential mechanism of action.

## 2. Results

### 2.1. Identification of ASs and Screening of 4HBA Safety and Efficacy in ASs

The immunofluorescence results showed high GFAP positivity in the cells and were consistent with the characteristics of primary ASs in the cortex; these could be used in subsequent experiments (Figure 1B). The cell viability results suggested that 4HBA did not affect the survival of normal ASs and had no inhibitory effect on their growth in the concentration range of 0–800 μmol/L (Figure 1C). Compared with that in the control group, the cell survival rate in the OGD/R group was noticeably diminished (*p* < 0.001). Compared with the OGD/R group, the 4HBA group had a markedly amplified cell survival rate (*p* < 0.05). When the concentration of 4HBA was 200 μmol/L, the improvement in AS cell survival was most significant (*p* < 0.001), followed by 100 μmol/L (*p* < 0.01) and 50 μmol/L (*p* < 0.05) (Figure 1D).

### 2.2. Effect of 4-HBA on CAT, MPTP, and Ca^2+^ Levels in ASs

**Figure 2:**
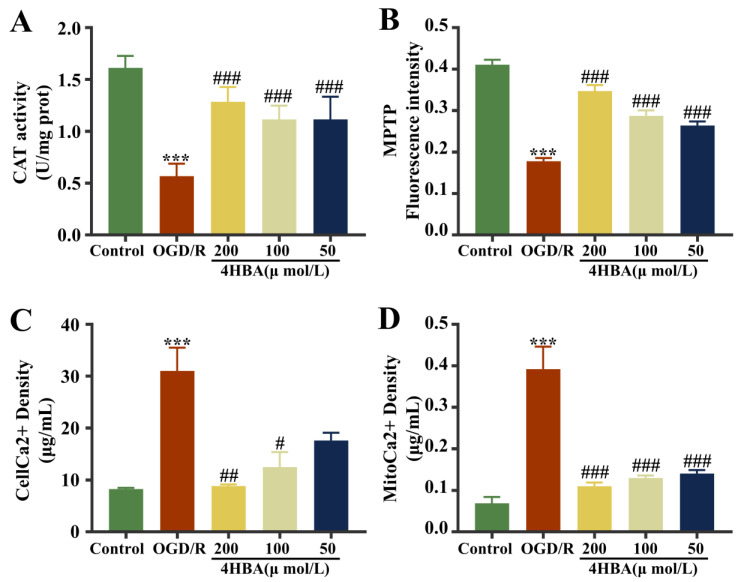
Effect of 4HBA on CAT activity (**A**), MPTP opening (**B**), and Ca^2+^ levels within cells (**C**) and mitochondria (**D**). Data are presented as the mean ± standard deviation (SD). ### *p* < 0.001, ## *p* < 0.01, # *p* < 0.05 vs. the control group. *** *p* < 0.001 vs. the OGD/R group. Abbreviations: OGD/R, oxygen–glucose deprivation/reoxygenation; 4HBA, 4-hydroxybenzyl alcohol; CAT, catalase; MPTP, mitochondrial membrane transition pore.

In the assay results, open MPTPs would result in the release of intracellular fluorescent dyes or be burst by extracellular substances. A decrease in fluorescence intensity suggests an increase in MPTP opening. Compared with the control group, the CAT activity and MPTP opening degree diminished markedly (*p* < 0.001), and the Ca^2+^ content in cells and mitochondria were noticeably amplified (*p* < 0.001) (Figure 2). The CAT activity and MPTP opening were markedly amplified (*p* < 0.001), and intracellular and mitochondrial Ca^2+^ levels were noticeably diminished (*p* < 0.001) after treatment with 4HBA at doses of 200, 100, and 50 μmol/L (Figure 2A,B,D). Intracellular Ca^2+^ levels diminished markedly at 200 μmol/L (*p* < 0.01) and 100 μmol/L (*p* < 0.05) of 4HBA. Ca^2+^ levels diminished at a 4HBA concentration of 50 μmol/L but were not noticeably different (Figure 2C). The combination of the effectiveness results suggested that the protective effect on ASs was strongest when the 4HBA concentration was 200 μmol/L. Therefore, a 200 μmol/L concentration of 4HBA was used for the subsequent experiments.

### 2.3. Effect of 4HBA on ROS Content in ASs

**Figure 3:**
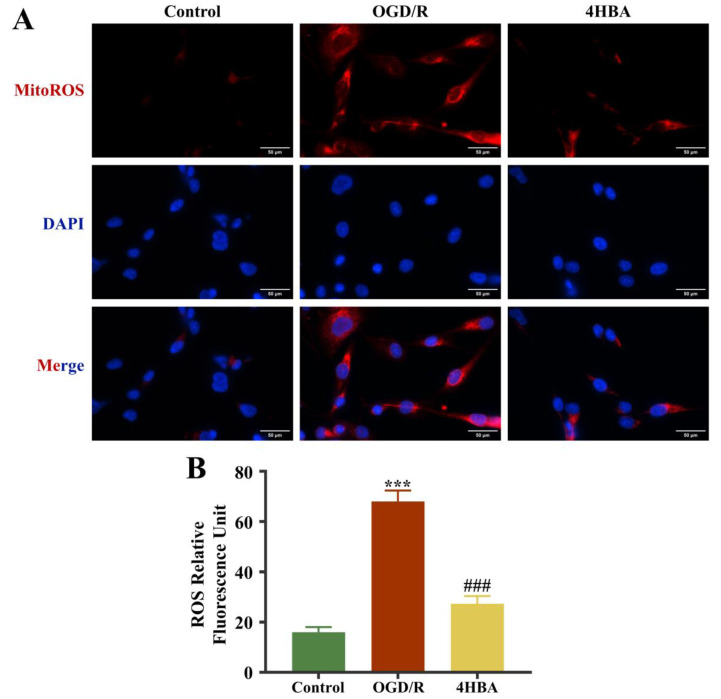
Immunofluorescence staining plots of ROS (bar = 50 μm) (**A**). Fluorescence intensity of ROS (**B**). Data are presented as the mean ± standard deviation (SD). ### *p* < 0.001 vs. the control group. *** *p* < 0.001 vs. the OGD/R group. Abbreviations: OGD/R, oxygen–glucose deprivation/reoxygenation; 4HBA, 4-hydroxybenzyl alcohol; ROS, reactive oxygen species.

The immunofluorescence results showed that MitoROS levels were markedly amplified in the OGD/R group compared with the control group (*p* < 0.001). 4HBA intervention resulted in a significant decrease (*p* < 0.001) in MitoROS levels (Figure 3).

### 2.4. Effect of 4HBA on Mitochondrial Morphology in ASs

**Figure 4:**
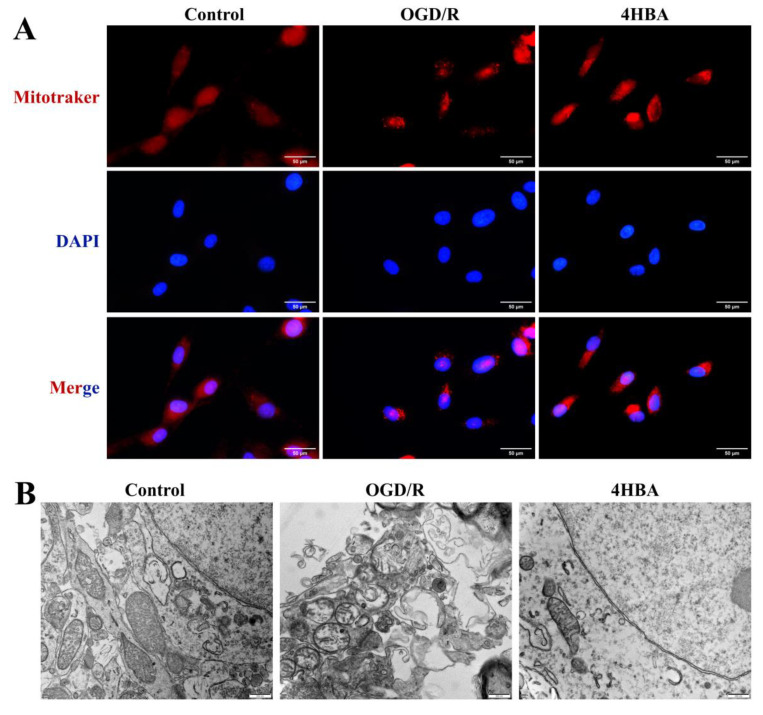
Immunofluorescence staining plots of MitoTracker (bar = 50 μm) (**A**). A representative picture of mitochondria in ASs by transmission electron microscopy (bar = 500 nm) (**B**). Abbreviations: OGD/R, oxygen–glucose deprivation/reoxygenation; 4HBA, 4-hydroxybenzyl alcohol.

From the results of MitoTracker fluorescence staining, it can be seen that mitochondrial punctate staining and broken mitochondrial morphology appeared more frequently in the OGD/R group compared with the control group. After 4HBA treatment, punctate staining was markedly reduced, and mitochondrial morphology was restored (Figure 4A).

In the electron microscopy results, mitochondria in the control group had a normal structure with clear membrane edges, relatively tight and intact cristae, and a uniform matrix. The mitochondria in the OGD/R group showed swelling and rupture, the membrane structure was disrupted, the cristae were sparsely broken, and the matrix was electronically shiny. After 4HBA treatment, the mitochondrial morphology was restored, the membrane tended to be intact, the matrix homogeneity was noticeably improved, and the ultrastructure was noticeably improved (Figure 4B).

### 2.5. Screening for Differential Metabolites in ASs

**Figure 5:**
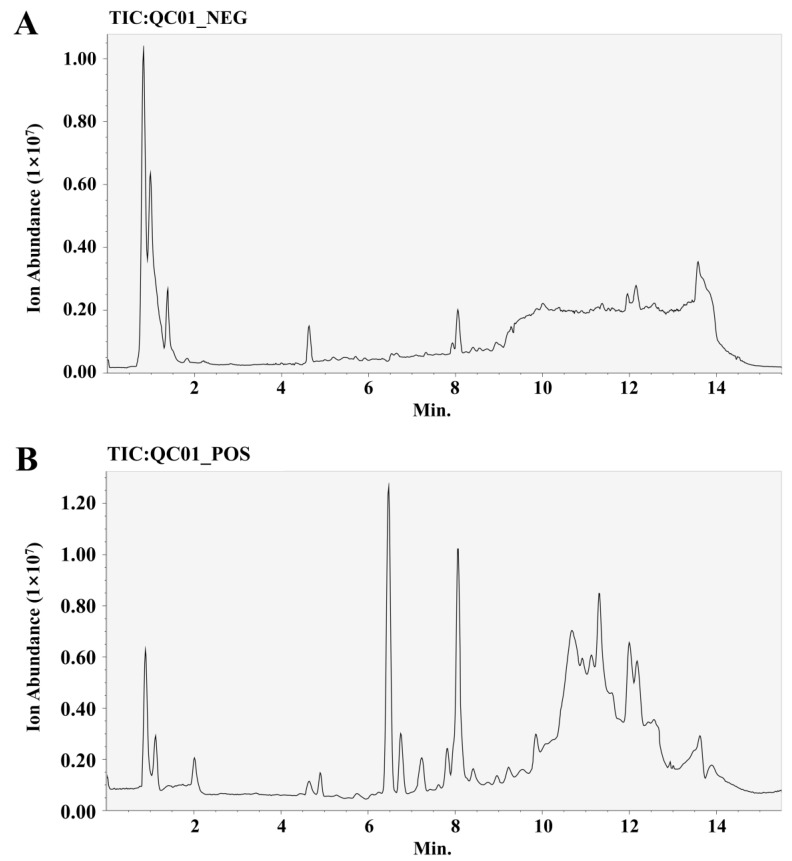
Typical chromatogram of negative ions (**A**). Typical chromatogram of positive ions (**B**). Abbreviations: NEG, negative; POS, positive.

**Figure 6:**
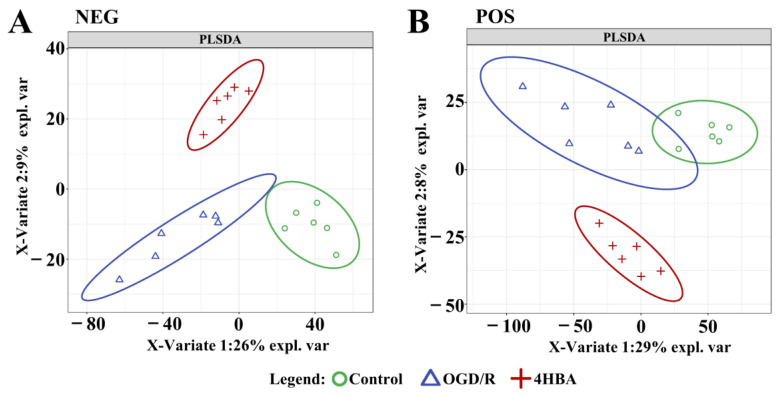
Enrichment analysis of differentially abundant metabolites between the control and OGD/R groups and 4HBA group PLSDA score map in positive mode (**A**). PLS-DA score diagram in negative mode (**B**). Abbreviations: NEG, negative; POS, positive; PLSDA, partial least squares discriminant analysis; 4HBA, 4-hydroxybenzyl alcohol.

**Figure 7:**
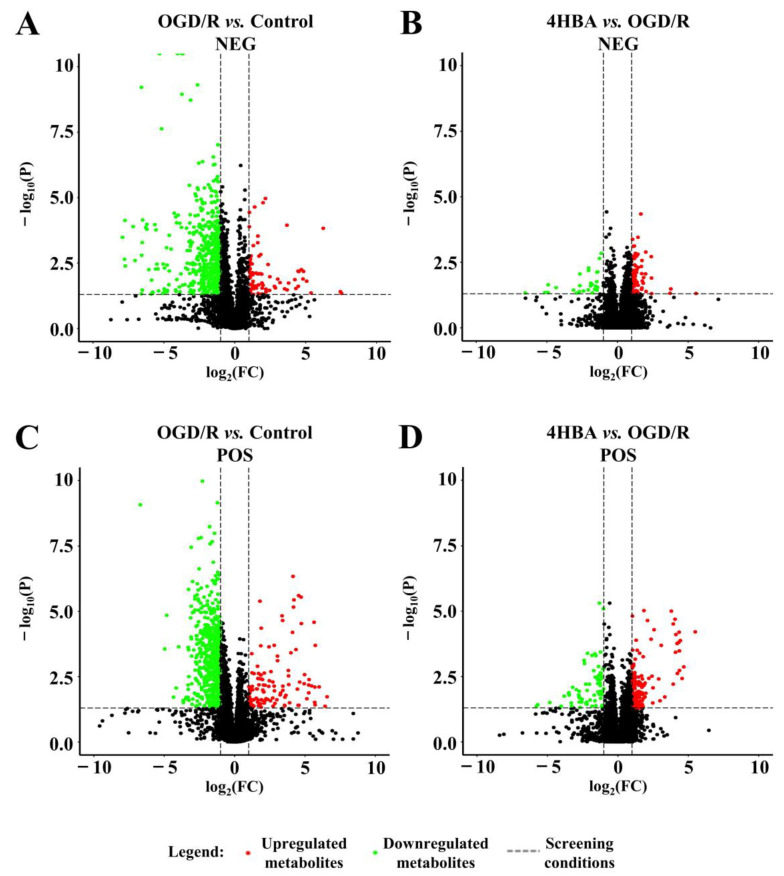
Volcano plot of the control and OGD/R groups in the negative ion model (**A**). Volcano plot of OGD/R and 4HBA in the negative ion model (**B**). Volcano plot of the control and OGD/R groups in the positive ion model (**C**). Volcano plot of OGD/R and 4HBA in the positive ion model (**D**). Abbreviations: NEG, negative; POS, positive; OGD/R, oxygen–glucose deprivation/reoxygenation; 4HBA, 4-hydroxybenzyl alcohol.

We collected each group of cells for metabolomics analysis. We performed feature peak extraction and preprocessing of LC-QTOF/MS data and organized the final results into a two-dimensional data matrix. A total of 3412 metabolites, including purines, pyrimidines, and amino acids, were identified in the MSDIAL database based on primary and secondary mass spectrometry information (Figure 5). To effectively differentiate the observations between groups, we established a supervised multidimensional PLS-DA model for statistical analysis. The results suggested that the metabolic profiles of the same groups achieved good clustering. The separation between the groups was obvious, indicating that the metabolites in the samples changed markedly after different treatments (Figure 6). In this experiment, the variable importance in the projection (VIP) value (threshold > 1) and *p* value from Student’s *t* test (threshold < 0.05) combined with the fold change (FC) value were used as screening conditions. The screening results are presented in the form of volcano plots; red dots represent metabolites with increased content and green dots represent metabolites with decreased content. In the positive ion mode, a total of 9 metabolites were upregulated and 51 metabolites were downregulated in the OGD/R group compared with the control group. Compared with OGD/R, 50 metabolites were upregulated and 11 metabolites were downregulated in the 4HBA group. In the negative ion mode, a total of 6 metabolites were upregulated and 27 metabolites were downregulated in the OGD/R group compared with the control group. Compared with OGD/R, 29 metabolites were upregulated and 4 metabolites were downregulated in the 4HBA group. A total of 82 differentially abundant metabolites were screened under positive and negative ion modes (Figure 7). There were 15 upregulated differential ion genes and 67 downregulated differential genes in the OGD/R group compared with the control group. The 4HBA group upregulated 69 differentially expressed genes and downregulated 13 differentially expressed genes compared to the OGD/R group. We used the FC values between the OGD/R group and the control group to sort from largest to smallest and took the top 20 versus the bottom 20 for presentation (Table 1). We chose cycloserine, which had the largest FC value in the OGD/R group compared to the control group and the smallest FC value in the 4HBA group compared to the OGD/R group, as the most noticeably different metabolite, for subsequent experiments.

### 2.6. KEGG Enrichment of Metabolic Pathways

**Figure 8:**
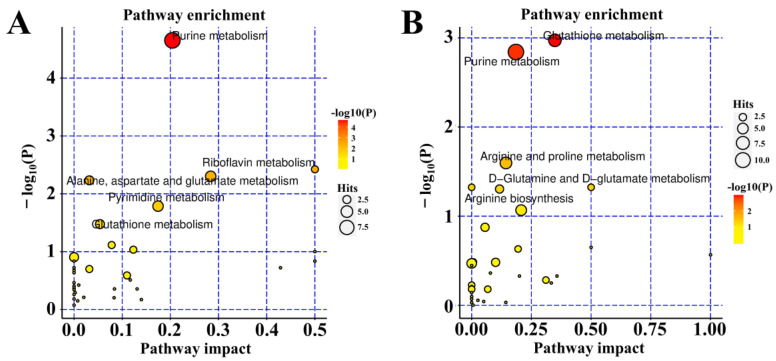
Pathway analysis of negative ion mode (**A**). Pathway analysis of positive ion mode (**B**).

To reflect the changes in the metabolic pathways of 4HBA in the treatment of OGD/R-injury ASs more intuitively, we localized the differentially abundant metabolites in the metabolic pathways for enrichment analysis. The results showed that the pathways mainly affected in the positive ion mode were glutathione metabolism, purine metabolism, and arginine and proline metabolism. In the negative ion mode, purine metabolism, pyrimidine metabolism, and glutathione metabolism were mainly affected (Figure 8).

### 2.7. Metabolomic Validation in ASs

**Figure 9:**
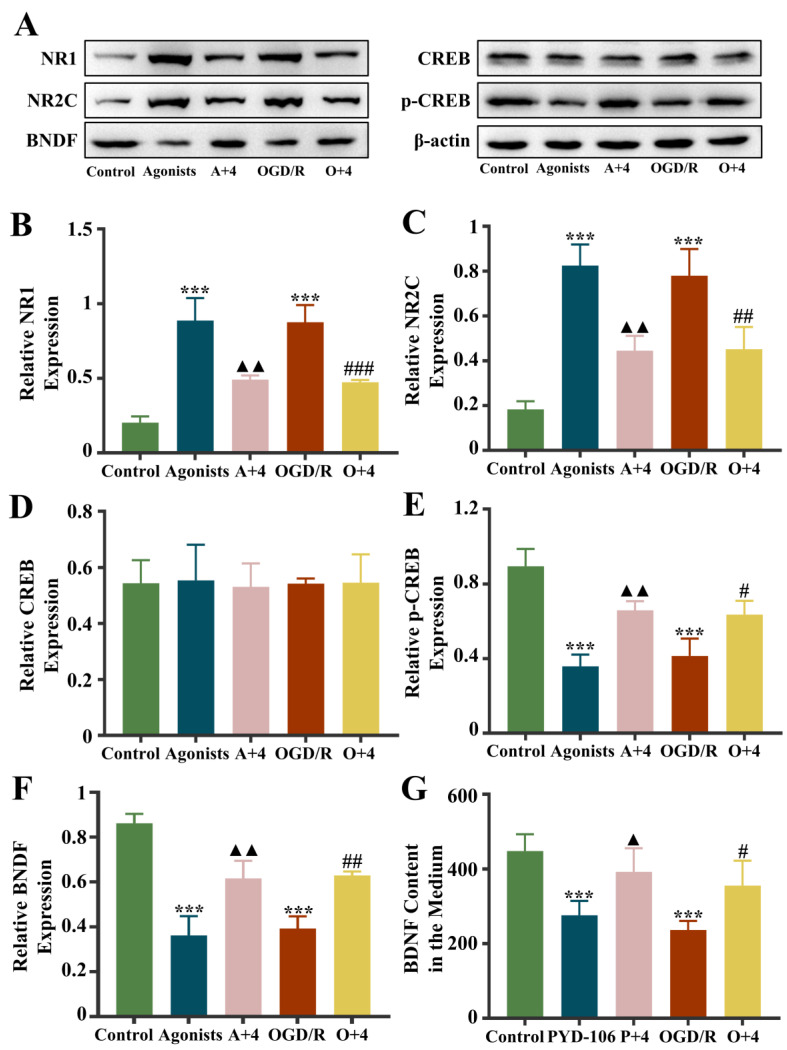
Fluorescence intensity of BDNF, NR1, NR2C, CREB, and p-CREB protein bands from the the control, agonist, A + 4, OGD/R, and O + 4 groups (**A**). Graphs showing the expression levels of NR1 (**B**), NR2C (**C**), CREB (**D**), p-CREB (**E**), and BDNF (**F**) in the five groups. The content of BDNF in the medium (**G**). Data are presented as the mean ± standard deviation (SD). *** *p* < 0.001 vs. the control group. ▲▲ *p* < 0.01, ▲ *p* < 0.05 vs. the agonist group, ### *p* < 0.001, ## *p* < 0.01, # *p* < 0.05 vs. the OGD/R group. Abbreviations: A + 4, agonist + 4HBA group; OGD/R, oxygen–glucose deprivation/reoxygenation; O + 4, OGD/R + 4HBA group.

Cycloserine elicits 190% of the current response in the NR1/NR2C receptors as glycine, while NR2C is specifically highly expressed in ASs. Therefore, we chose the NR1/NR2C receptor as the entry point and added the NR1/NR2C receptor agonist PYD-106 for metabolomics validation studies (Figure 9). The western blotting results showed that the expression of NR1 and NR2C was markedly higher in the agonist group than in the control group and the OGD/R group (*p* < 0.001), and the expression of BDNF and p-CREB was noticeably diminished (*p* < 0.001). Compared with the agonist group, NR1 and NR2C expression was markedly diminished in the agonist + 4HBA group (*p* < 0.01), and BDNF and p-CREB expression was noticeably amplified (*p* < 0.01). Compared with the OGD/R group, NR1 (*p* < 0.001) and NR2C (*p* < 0.01) expression was markedly diminished, and BDNF (*p* < 0.01) and p-CREB (*p* < 0.05) expression was noticeably elevated in the OGD/R + 4HBA group. In the detection of BDNF content in the medium, the BDNF content in the agonist group and the OGD/R group diminished markedly (*p* < 0.001), and the BDNF content was markedly elevated (*p* < 0.05) after 4HBA treatment. It is suggested that 4HBA may exert a protective effect on ASs by improving the NR1/NR2C-BNDF-CREB pathway.

### 2.8. Effect of 4HBA on BBB Exudation in 2VO Rats

**Figure 10:**
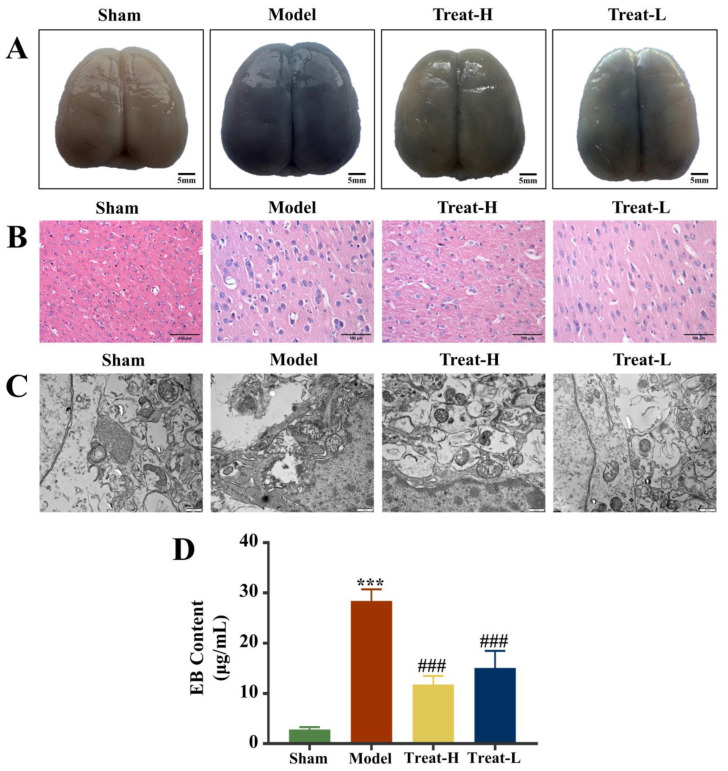
EB staining diagram (bar = 5 mm) (**A**) and HE staining diagram (bar = 100 μm) (**B**) of each group of brain tissues. Electron microscopy images of mitochondria (bar = 500 nm) (**C**) and EB content (**D**) of brain in each group. Data are presented as the mean ± standard deviation (SD). *** *p* < 0.001 vs. the sham group. ### *p* < 0.001 vs. the model group. Abbreviations: EB, Evans blue.

Compared with the sham group, the Evans blue (EB) content in the brain tissue of the model group was noticeably amplified (*p* < 0.01) and, after 4HBA treatment, the EB contents in the Treat-H group and the Treat-L group diminished noticeably (*p* < 0.01, Figure 10A,D).

### 2.9. Effect of 4HBA on Brain Tissue and Mitochondria in 2VO Rats

Under HE staining, the brain tissue of the sham group showed a standard healthy state, with intact structure, orderly cell arrangement, intact morphology, and clear nuclei, and no abnormalities were observed. Comparatively, the brain tissue of the rats in the model group showed significant pathological changes, which manifested as tissue edema, enlarged intercellular space, cell body lysis, nuclear condensation and fragmentation, and chaotic arrangement of the nerve fibers. After the treatment with 4HBA, the brain histopathological condition of the rats in the Treat-H group and the Treat-L group was markedly improved. The treatment alleviated the abnormal changes in the morphology of brain cells and noticeably restored the normal structure of brain tissue (Figure 10B).

Electron microscopy further demonstrated the good condition of mitochondria in the sham group, with normal mitochondrial appearance, intact membrane structure, and uniform matrix staining, reflecting the normal function of mitochondria. Conversely, the mitochondria of the model group suffered severe damage, which was manifested by shrinkage of membrane structure, amplified fissures, and abnormal matrix electron density. After treatment, the mitochondrial ultrastructure of the rats in the Treat-H and Treat-L groups was markedly restored, the morphology and membrane integrity were repaired, the matrix density was reduced, and most mitochondria remained intact (Figure 10C).

### 2.10. Effect of 4HBA Connexin Protein in 2VO Rats

**Figure 11:**
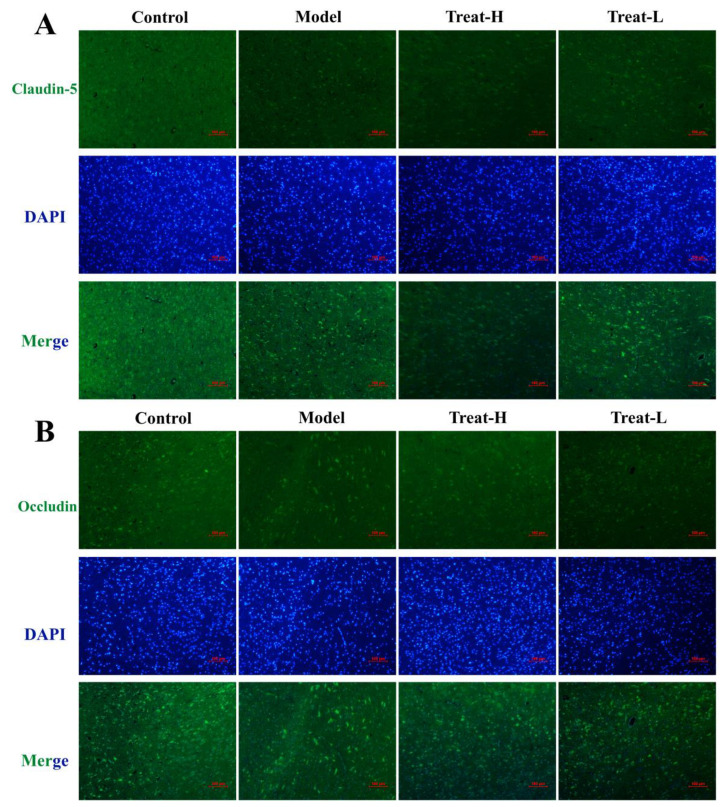

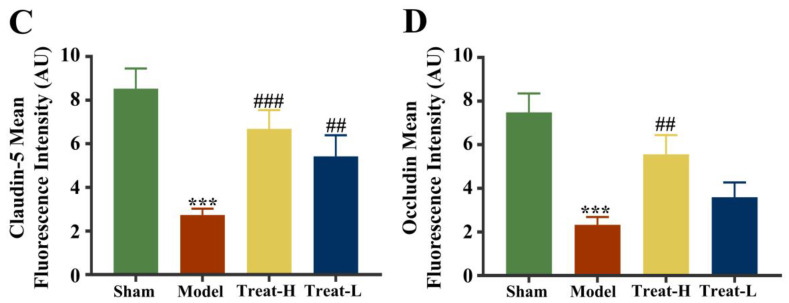
Claudin-5 (**A**) and Occludin (**B**) fluorescence expression patterns, and Claudin-5 (**C**) and Occludin (**D**) fluorescence average optical density in each group (bar = 100 μm). Data are presented as the mean ± standard deviation (SD). *** *p* < 0.001 vs. the sham group. ### *p* < 0.001, ## *p* < 0.01 vs. the model group.

**Figure 12:**
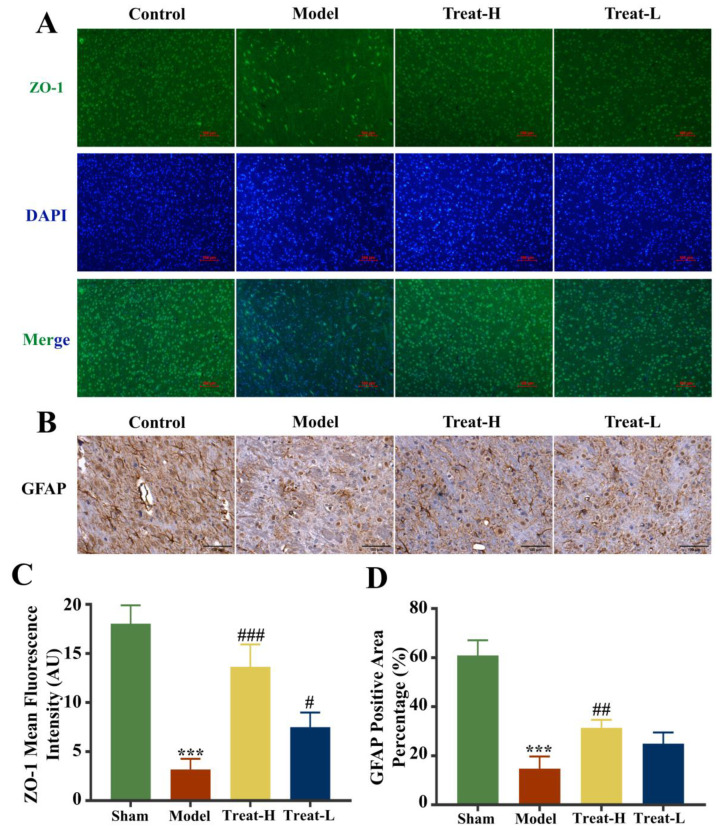
ZO-1 fluorescence expression patterns (bar = 100 μm) (**A**), GFAP immunohistochemistry images (bar = 100 μm) (**B**), ZO-1 fluorescence average optical density (**C**), and GFAP positive area percentage (**D**) in each group. Data are presented as the mean ± standard deviation (SD). *** *p* < 0.001 vs. the sham group. ### *p* < 0.001, ## *p* < 0.01, # *p* < 0.05 vs. the model group.

After the replication of the 2VO model, the expression of Claudin-5, Occludin, and ZO-1 in the model group diminished noticeably compared with that of the sham group (*p* < 0.001). After 4HBA administration, the expression of Claudin-5, ZO-1 (*p* < 0.001), and Occludin (*p* < 0.01) in the Treat-H group was markedly amplified. The expression of Claudin-5 (*p* < 0.01) and ZO-1 (*p* < 0.05) was noticeably amplified in the Treat-L group. The expression of Occludin recovered, but the difference was not statistically significant (Figure 11 and Figure 12A,C).

### 2.11. Effects of 4HBA on the Expression of GFAP, AQP4 and BDNF in 2VO Rats

**Figure 13:**
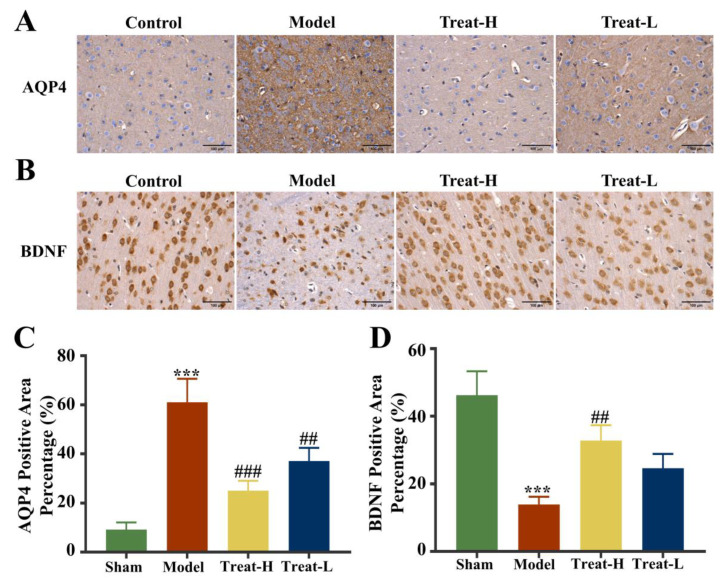
AQP4 (**A**) and BDNF (**B**) immunohistochemistry images (bar = 100 μm), and AQP4 (**C**) and BDNF (**D**) positive area percentage in each group. Data are presented as the mean ± standard deviation (SD). *** *p* < 0.001 vs. the sham group. ### *p* < 0.001, ## *p* < 0.01 vs. the model group.

Compared with the sham group, the expression of GFAP and BDNF diminished markedly (*p* < 0.001) and AQP4 was markedly amplified (*p* < 0.001) in the model group. In the Treat-H group, the expression of GFAP and BDNF were markedly amplified (*p* < 0.01); AQP4 also diminished remarkably (*p* < 0.001). GFAP and BDNF in the Treat-L group were amplified but the difference was not statistically significant, while the expression of AQP4 diminished markedly (*p* < 0.01, Figure 12B,D and Figure 13).

### 2.12. Effect of 4HBA on NR1/NR2C-CREB-BNDF Pathway in 2VO Rats

**Figure 14:**
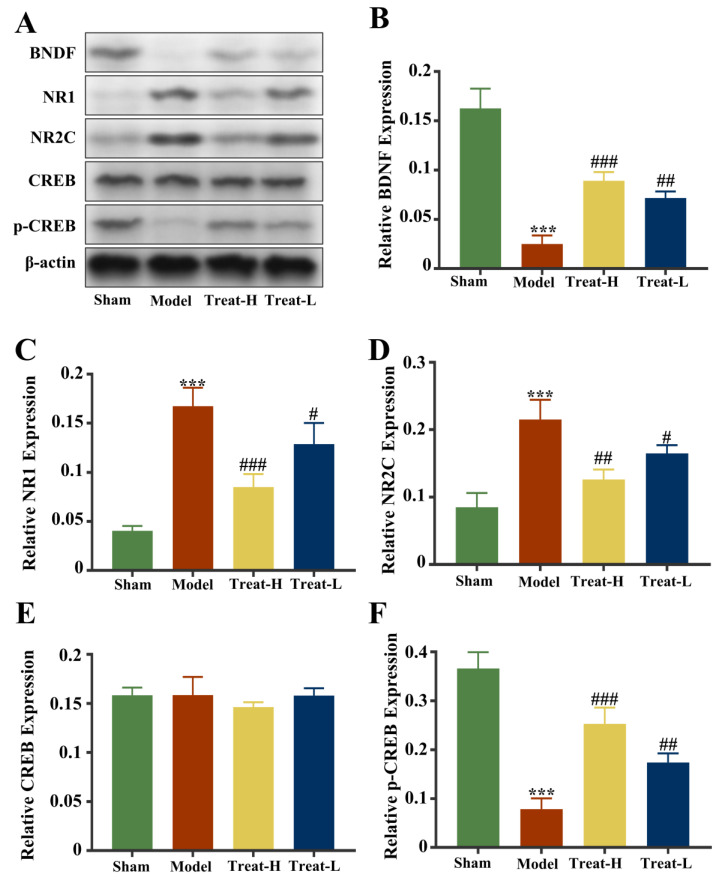
Fluorescence intensity of BDNF, NR1, NR2C, CREB, and p-CREB protein bands from each group (**A**). Graphs showing the expression levels of BDNF (**B**), NR1 (**C**), NR2C (**D**), CREB (**E**), and p-CREB (**F**) in the four groups. Data are presented as the mean ± standard deviation (SD). *** *p* < 0.001 vs. the sham group. ### *p* < 0.001, ## *p* < 0.01, # *p* < 0.05 vs. the model group.

Compared with the sham group, the expression of NR1 and NR2C in the model group was markedly amplified (*p* < 0.001), while the expression of p-CREB and BDNF diminished (*p* < 0.001). After 4HBA treatment, the expression of NR1 (*p* < 0.001) and NR2C (*p* < 0.01) in the Treat-H group diminished noticeably, while the expression of p-CREB and BDNF was amplified (*p* < 0.001). The expression of NR1 and NR2C in the Treat-L group diminished (*p* < 0.05), while the expression of p-CREB and BDNF was amplified (*p* < 0.01, Figure 14).

## 3. Discussion

We found that the cerebral ischemia in vitro results showed a significant decrease in AS cell viability. In addition, in vivo experiments also showed edema and connection damage in ASs. After 4HBA treatment, the viability of ASs increased significantly, and the edema and damage were significantly reduced. ASs play multiple roles, such as transmitting information, providing nutrition, and regulating the function of the blood–brain barrier [27]. It is worth noting that when cerebral ischemia occurs, ASs are extremely resilient and adaptive in the anaerobic environment due to the large amount of glycogen stored in them [28]. At the same time, they utilize their advantages to supply energy to neurons, repair damaged neurons, and further regulate homeostasis in ischemic areas within the brain [29]. Therefore, ASs are the key cells that maintain homeostatic balance in the brain [30].

To elucidate the changes in substance metabolism caused by 4HBA’s action on OGD/R-injured ASs, we performed metabolomics analysis. The results showed that the differentially metabolized substances involved in the 4HBA intervention process contained purines, pyrimidines, and amino acids. Notably, azelaic acid levels appeared elevated at the onset of OGD/R injury and the levels diminished markedly after 4HBA treatment. Elevated azelaic acid is indicative of enhanced fatty acid oxidation in a variety of brain diseases, including cerebral ischemia, and has been used as a disease biomarker [31]. Glutathione (GSH), cysteinylglycine (Cys), ADP, and N-acetylglutamate levels were noticeably diminished in the OGD/R group compared to the control group, and these levels were markedly restored after 4HBA treatment. GSH plays an important role in the regulation of antioxidant defense, nutrient metabolism, and cellular events [32]. GSH deficiency leads to oxidative stress, which plays a key role in the pathogenic mechanisms of CIRI injury sites [33]. Metabolic interactions between neurons and ASs are critical for GSH synthesis [34]. In vitro experiments performed in cocultured neurons and ASs showed that GSH levels in neurons were amplified in the presence of ASs [35]. Membrane-associated enzymes on the surface of ASs convert extracellular GSH to the dipeptide CysGly and subsequently to Cys and Gly [36]. GSH and Cys were markedly diminished in the experimental results, suggesting that OGD/R may inhibit GSH metabolism. Meanwhile, ADP is an important substrate for ATP generation [37]. 4HBA can generate energy for ASs to counteract OGD/R injury by increasing ADP content. N-Acetylglutamic acid also has an anti-ischemic effect, which can improve CIRI, and thus, induce anti-ischemic effects [38]. The enrichment results suggested that these differentially abundant metabolites are mainly involved in purine metabolism, pyrimidine metabolism, and GSH metabolism; this suggested that nucleotide metabolism and oxidative stress-related metabolism play an important role in the protection of OGD/R-injured ASs by 4HBA. GSH metabolism is an important pathway related to oxidative stress [39]. The levels of GSH and pyroglutamate within the pathway diminished in the OGD/R group compared with the control group, and their levels rebounded after 4HBA treatment, suggesting that 4HBA has significant potential in correcting oxidative stress to counteract OGD/R injury.

The metabolomics results also indicated that cycloserine, the metabolic differentiator with the most significant differences, had an FC value of 44.5 for the control group vs. the OGD/R group, i.e., cycloserine was 44 times more abundant in the OGD/R group than in the control group. In contrast, the FC value was only 0.14 for Treat vs. OGD/R, i.e., the amount of cycloserine within the 4HBA group was only 14% of the amount in the model group. Therefore, we selected cycloserine as a significant metabolic differentiator for validation. Cycloserine is a partial subunit super agonist of the N-methyl-d-aspartate (NMDA) receptor [40].

The common subunit combinations of NMDA are mainly composed of NR1, NR2 (2A, 2B, 2C, 2D), NR3, etc. [41]. The different subunit compositions are the key to the diversity in bioactivities expressed by NMDA [42]. NMDA is a key trigger to initiate apoptosis during the acute phase of cerebral ischemia [43]. Some experimental studies have shown that cycloserine elicits 190% of the current response at NR1/NR2C receptors as glycine [44]. This is extremely detrimental to ASs in the brain when it occurs during the acute phase of cerebral ischemia because NR2C is highly expressed in ASs in cortical regions of the brain and is absent from neurons and glial cells [45]. The strong activation of NMDA by cycloserine via NR1/NR2C leads to a large inward Ca^2+^ flow, resulting in calcium overload and the activation of a series of downstream pro-death signals, which induces apoptotic events [46]. The cell death dilemma brought about by cerebral ischemia is exacerbated in this process [47]. It has been shown that NR2C-knockout mice have a 50–60% reduction in cerebral infarct volume compared to wild-type mice, attenuate cerebral edema, and express a strong and significant nerve recovery function by modifying the NMDA/p-CREB cell survival signaling axis [48]. Brain-derived neurotrophic factor (BDNF) is one of the proteins upregulated by CREB-dependent pathways in ASs [49]. The release of BDNF is one of the important roles of ASs in protecting neighboring neurons, and BDNF is released from ASs through cytokinesis to regulate the function of neighboring neurons [50].

PYD-106 is a stereoselective pyrrolidone (PYD) orthosteric modulator that markedly enhances NR1/NR2C receptor responses [51]. Therefore, we performed metabolomics validation experiments using PYD-106 as an NMDA-selective agonist. We found by observational western blotting experiments that when the agonist was added to normal cultured cells and when the model replication was successful, NR1/NR2C expression was noticeably elevated and BDNF and p-CREB expression was noticeably diminished, consistent with the OGD/R trend. 4HBA treatment markedly corrected this set of deviations. This result suggests that 4HBA may address the apoptotic dilemma of cerebral ischemic ASs and their peripheral neurons by specifically repairing the NMDA/p-CREB/BNDF survival signaling axis. This was also verified by us in in vivo experiments.

BDNF also exerts protective effects against the apoptosis of neuronal cells caused by oxidative stress and impaired mitochondrial activity under ischemic conditions. Excessive intracellular Ca^2+^ in ASs also leads to amplified ROS production, and diminished CAT activity is not able to remove excessive ROS in time, resulting in cellular oxidative stress injury [52]. Meanwhile, it has been shown that mitochondria undergo persistent swelling after a few minutes of NMDA overactivation, and this injury may be related to the influx of large amounts of Ca^2+^ targeting the mitochondria [53]. The continuous opening and swelling of MPTP causes mitochondrial dysfunction, which further aggravates ischemic brain cell injury [54]. Therefore, calcium overload after NMDA overactivation not only triggers the inactivation of cAMP response element-binding protein (CREB), which leads to the downregulation of BDNF levels, but also induces cellular oxidative stress injury and mitochondrial dysfunction [55,56]. Relevant indices showed that 4HBA was able to effectively attenuate the intracellular and mitochondrial Ca^2+^ overload induced by OGD/R injury and reduce ROS content. Meanwhile, elevating CAT activity and attenuating the opening of the MPTP transition pore could counteract the oxidative stress and mitochondrial dysfunction injury triggered by CIRI.

We further explored the protective effect of 4HBA on ASs in vivo by replicating the 2VO rat model to simulate the acute ischemic chronic hypoperfusion state. GFAP, as the signature link protein of ASs, is able to provide stability and mechanical strength to maintain cell morphological stability [57]. AQP4 is also highly expressed in ASs and plays an important role in regulating water molecule transport, supporting cell function, and neuroprotection and repair [58]. However, its overactivation exacerbates the formation of cerebral edema, causing further damage to brain tissue [59]. BDNF is the most important product of ASs, affecting the normal work of peripheral neuronal cells [60]. 4HBA could effectively restore the content of GFAP and BDNF and reduce the overactivation of AQP4, suggesting that 4HBA could markedly protect ASs in 2VO model rats.

Based on the important position of ASs in the BBB of the brain, we also explored the protective effect of 4HBA on the BBB in vivo. When brain damage occurs, albumin, a liver-specific product, penetrates into the brain tissue through blood vessels due to BBB destruction. EB is a dye that binds specifically to albumin, and the degree of staining reflects the integrity of the BBB [61]. We found that after the successful replication of the 2VO model, the EB content in the brain was amplified, indicating that the BBB permeability was amplified and the integrity was damaged, while after 4HBA treatment, the BBB was markedly repaired and the EB content in the brain diminished. Claudin-5 and Occludin are linked to each other through their transmembrane domains, while ZO-1 interacts with these transmembrane proteins and cytoskeletal components through their cytosolic domains, and they work together to maintain tight junctions between cells, thereby maintaining the normal function of the BBB [62,63]. When cerebral ischemic injury occurs, the expression of these links in brain tissue decreases markedly, suggesting that the tight junction of the BBB is severely damaged. The expression of connexin rebounded after 4HBA administration, indicating that 4HBA plays a crucial role in stabilizing BBB tight junctions.

## 4. Materials and Methods

### 4.1. Animal

Sprague Dawley rats, 0–3 days old, weighing 7–9 g, were provided by Cavins Laboratory Animal Co., Ltd., Changzhou, China (Animal Qualification Certificate No.: SCXK (Su) 2022-0010). Forty SPF-grade Sprague Dawley rats, 6–8 weeks old, weighing 250 g–280 g, were provided by the Department of Animal Experiments, Kunming Medical University, Kunming, China (Animal Qualification Certificate No.: SCXK(Dian)2022-0004). The rats were housed in an SPF-grade environment at 22 ± 2 °C and fed and watered ad libitum. All animal experiments were approved by the Animal Ethics Committee of the Yunnan University of Traditional Chinese Medicine, Yunnan, China (Number. R-062019039, Date: March 2019), and the care and use of experimental animals were carried out in accordance with the guidelines of the American National Institutes of Health. All efforts were made to reduce the number and the suffering of the animals.

### 4.2. Drugs and Cell Culture Conditions

4HBA was purchased from ABPHYTO Co. (Chengdu, China, Item No. AB0478). The complete medium was prepared with a ratio of 1% penicillin–streptomycin liquid (C3420-0100, VivaCell Biosciences Ltd., Shanghai, China), 10% exosome-free FBS medium (BS-1101S, Opcel Biotechnology Co., Ltd., Inner Mongolia, China), and 89% DMEM high-sugar medium (BC-M-038, BioChannel Biological Technology Co., Ltd., Nanjing, China). The medium was used for daily cell culture and experimental manipulation. Cells were cultured at 37 °C in a 5% CO_2_ incubator (HF90HT, Lishen Scientific Equipment Co., Ltd., Shanghai, China).

### 4.3. Extraction of Rat Cortical Primary ASs

Suckling rats 3 days of age were sterilized using 75% alcohol, and the heads of the suckling rats were quickly severed and placed in HBSS dissection solution (YS180323, Yaji Biological Technology Co., Ltd., Shanghai, China). Intact brain tissue was removed on ice and placed in HBSS solution containing 1% penicillin–streptomycin liquid. After stripping the surface meninges and blood vessels under the microscope (SZ650BP, Optec Instrument Co., Ltd., Chongqing, China), the brain tissues were cut into 1 mm^3^ tissue blocks. The tissue was digested with 0.25% trypsin (C3530-0500, VivaCell Biosciences Ltd., Shanghai, China) at 37 °C for 10 min. The digestion was terminated with an equal volume of complete medium and blown until no tissue mass was present. After centrifugation at 1000× *g* for 10 min (cellsmart, Copyright DHS Life Science & Technology Co., Ltd., Beijing, China), the cells were inoculated in Petri dishes. Differential adhesion treatment was repeated twice for 1 h each time. After filtering with a 74 μm pore size cell sieve, the cells were centrifuged at 1000× *g* for 10 min. The cells were inoculated into new culture flasks. After 80% of the cells had fused, cultured rat cortical primary ASs were taken and identified using immunofluorescence for glial fibrillary acidic protein (GFAP) expression (Anti-GFAP antibody #3670, Cell Signaling Technology, Boston, MA, USA). The following experiments were performed after identification.

### 4.4. Drug Administration and Model Replication in Groups

In vitro, the experiment was divided into the control group, OGD/R group, and 4HBA group. The 4HBA group was administered treatment throughout the 24 h period before modeling to the end of the reoxygenation session. The control group and OGD/R group media were synchronously replaced with drug-free complete medium. Except for the control group, the groups were incubated in serum-/glucose-free medium (11966025, Thermo Fisher Scientific, Pittsburgh, PA, USA) pre-injected with oxygen-deficient gas (95% N_2_ + 5% CO_2_) for 30 min, and then, incubated in a closed incubator with hypoxia for 6 h. The replication of the OGD/R model was completed after replacement with complete culture based on 24 h of incubation under normal culture conditions.

In vivo, the experiment was divided into the sham group, model group, and Treat-H and Treat-L groups. According to the preliminary results of the research group, 20 mg/kg was used as the high dose of 4HBA and 10 mg/kg was used as the low dose of 4HBA. The rats in the sham group and model group were given equal amounts of normal saline. After 7 days of continuous gavage, the groups other than the sham group were used to replicate the 2VO acute ischemic sustained hypoperfusion model. The rat bilateral common carotid arteries were isolated, ligated, and sheared. The rats in the sham group had both common carotid arteries isolated and no other treatments were performed. Dosing was continued for 30 days after the replication of the model, and brain tissue was collected as a sample for subsequent experiments.

### 4.5. Cell Viability Assay for Drug Safety and Efficacy

ASs were inoculated in 96-well plates at a density of 9 × 10^4^. A blank group with no cells was established by adding complete medium only. A mass of 0.25 g of MTT powder (M8180, Solarbio Science & Technology Co., Ltd., Beijing, China) was volume reduced to 50 mL of PBS and prepared into a final concentration of 5 mg/mL of MTT solution. Referring to Section 2.4 for drug administration and replication of the model, 100 μL of 5 mg/mL MTT solution was added to each well at the end of the reoxygenation session. The cells were incubated for 4 h in an incubator under normal conditions and protected from light. After incubation, the supernatant was discarded, and 150 μL of DMSO solution was added and shaken for 15 min to fully dissolve the crystals. The OD value was measured at 490 nm using an VariosKan Flash enzyme-labeling instrument (5250040, Thermo Fisher Scientific, Pittsburgh, PA, USA).

Cell survival rate (%) = (A experimental group − A blank group)/(A control group − A blank group).

### 4.6. Detection of Intracellular CAT, MPTP, and Ca^2+^ Content in ASs and Ca^2+^ Content in Mitochondria

Cells were seeded at a density of 8 × 10^4^ in 6-well plates. Refer to Section 2.4 for administration and model replication after the end of the reoxygenation session. CAT (AKAO003-1M, Boxbio Science & Technology Co., Ltd., Beijing, China), MPTP (KTA4002, Abbkine, Inc., Wuhan, China), and Ca^2+^ colorimetric assay kits (S1063S, Beyotime Institute of Biotechnology, Beijing, China) were used according to the operating instructions to determine the AS intracellular CAT, MPTP, and Ca^2+^ contents. After extracting high concentrations of mitochondria using a mitochondrial extraction kit (EX2610, Beijing Solarbio Science & Technology Co., Ltd., Beijing, China), a Ca^2+^ colorimetric assay kit was used to detect the Ca^2+^ content in mitochondria according to the manufacturer’s instructions.

### 4.7. Flow Cytometry to Detect Mitochondrial Membrane Potential

Species were seeded at a density of 8 × 10^4^ in 6-well plates. Refer to Section 2.3 for dosing and replication of the model; after the re-glycosis and reoxygenation link, the cells were trypsinized with 0.25% without EDTA, centrifuged at 1200× *g*, collected for 5 min, washed twice with PBS, and resuspended with medium to make the number of cells 1 × 10^6^. Then, 1 μL of 50 mM CCCP solution was added to the control tube and it was incubated in a normal incubator for 5 min. Following this, 10 μL of 200 μM JC-1 working solution was added and incubated at 37 °C for 20 min. Then, 2 mL of PBS was used to wash the cells and they were centrifuged to remove the supernatant. After adding 500 μL of PBS to resuspend the cells, mitochondrial MMPs were analyzed using flow cytometry.

### 4.8. MitoTracker Red Staining to Measure Changes in Mitochondrial Morphology

Cells were seeded at a density of 2 × 10^5^ in confocal Petri dishes. Refer to Section 2.4 for administration and model replication. At the end of the reoxygenation session, 2 mL of PBS was used to wash the cells twice. MitoTracker™ Red FM (M22425, Thermo Fisher Scientific, Pittsburgh, PA, USA) staining working solution with a final concentration of 40 nM preheated at 37 °C was added and incubated for 30 min. At the end of the incubation, the cells were washed 3 times with serum-free medium, and the cell suspension was mixed thoroughly. The cells were placed under a Zeiss LSM laser confocal microscope (Zeiss, Oberkochen, Germany) for observation and photography.

### 4.9. MitoROS Levels Detected by Mitochondrial Superoxide Red Fluorescent Probe

Cells were seeded at a density of 2 × 10^5^ in confocal Petri dishes. Refer to Section 2.4 for administration and model replication. After the reoxygenation session, the cells were washed 3 times using PBS. Then, 2 mL of 5 μM MitoSOX™ Red (M36008, Thermo Fisher Scientific, Pittsburgh, PA, USA) reagent working solution was added and incubated for 10 min at 37 °C away from light. After washing 3 times with PBS solution, the cells were observed under a laser confocal microscope.

### 4.10. Metabolomics Assay

ASs were inoculated in 6-well plates with 8 × 10^4^ cells per well. The cells were divided into the OGD/R group and 4HBA group (OGD/R + 4HBA), and all 2-well cells were collected as 1 sample. The samples were sent to Nanjing Boon Biotechnology Co. for metabolomics assays. The cells were lysed by adding 100 μL of ultrapure water to the cell precipitates by repeated freezing and thawing 3 times. Next, 300 μL of methanol was added, shaken well for 5 min, and centrifuged at 18,000× *g* for 10 min. Then, 300 μL of supernatant was taken, and 100 μL of a mixture of ultrapure water and methanol (1:1, *v*/*v*) was added after evaporating to dryness. After centrifugation at 18,000× *g* for 10 min, the supernatant was collected. The samples were analyzed using Sciex Triple TOF 5600+ LC-QTOF/MS (AB Sciex Pte. Ltd., Shanghai, China) with 5 μL injection per sample. The UPLC conditions were as follows: the column was a Waters HSS T3 1.8 μm, 2.1 × 100 mm column. The column temperature was 40 °C, and the flow rate was 0.3 mL/min. Mobile phase A was the aqueous phase (ultrapure water + 0.1% formic acid), and mobile phase B was the organic phase (acetonitrile). The elution program was set as 95% A + 5% B (1.5 min), 85% A + 15% B (2.5 min), 40% A + 40% B (6 min), 5% A + 95% B (10 and 12 min), and 95% A + 5% B (12.5 and 15.5 min). Mass testing was performed in positive and negative ion modes, respectively, and scans were analyzed using Turbo V electrospray ionization (ESI). The parameters were set as follows: ion spray voltage: 7 kV; turbo spray temperature (TEM): 550 °C; declusterization potential (DP): 70 V; collision energy (CE): 30 eV; nebulizer gas (gas 1): 55 psi; heater gas (gas 2): 55 psi; curtain gas: 35 psi. The nebulizer and auxiliary gases were held by nitrogen. The scanning range of the TOF MS was *m*/*z* 50–1200 Da. The response intensities of each substance in all samples were modeled to discriminate and then analyze the data. In order to verify the quality control samples and preliminarily determine whether there were differences between groups, principal component analysis was performed. We used the R software (R project, version 4.2.1) to normalize the data groups in the standard analysis to obtain more intuitive and reliable results. A supervised multidimensional statistical approach, i.e., partial least squares determinant analysis (PLS-DA) model construction, was used to obtain the values of differentially expressed metabolites and variable importance in the projection (VIP). Metabolites were identified in the MSDIAL database based on primary and secondary mass spectrometry information. Next, *p* values were obtained using Student’s *t* test. VIP > 1, *p* < 0.05, and fold change (FC) values were used to screen differentially expressed metabolites. Finally, we enriched the relevant metabolic pathways of differentially expressed metabolites using the KEGG database (http://www.genome.jp/kegg/pathway.html, accessed on 5 April 2022). The enriched pathways were analyzed using MetaboAnalyst (https://www.metaboanalyst.ca/, accessed on 5 April 2022).

### 4.11. BDNF Content in Culture Medium Was Detected

Cells were seeded in confocal Petri dishes at a density of 2 × 10^5^; refer to Section 2.3 for dosing and replication. After the rehydration and reoxygenation process, the content of BDNF in the medium was detected using an ELISA kit (RA20017, Bioswamp Bio-Technology Co., Ltd., Wuhan, China).

### 4.12. EB Experiment

Rats were injected with 2% EB dye 4 mL/kg (O28HS199250, yuanye Bio-Technology Co., Ltd., Shanghai, China) via the femoral vein. After staining for 2 h, rats were deeply anesthetized with 1% sodium pentobarbital (catalog number: 57-33-0, Victor Biotechnology Co., Ltd., Chengdu, China) 40 mg/kg, and normal saline was perfused from the left ventricle to remove excess EB dye. After clear saline was perfused, the intact brain tissue of the rat was removed, photographed, and the EB dye content was measured.

### 4.13. HE Staining

Brain tissue samples were made into paraffin sections and dried, deparaffinized with xylene, hydrated with ethanol at gradient concentrations, and washed with distilled water. The nucleus was stained with hematoxylin for 2 min, differentiated with hydrochloric acid alcohol for a few seconds, and washed back to blue. Eosin staining solution was applied for 1 min, washing with water to rinse off the residual staining solution (KGA224, KGI Biotechnology Co., Ltd., Jiangsu, China). The sections were dehydrated and dried using ethanol at gradient concentrations; xylene made them transparent, and the neutral gum was sealed. A field of view was randomly selected using a phase-contrast microscope and pictures were taken using a 400× field of view.

### 4.14. Immunofluorescence Detection

Brain paraffin sections were deparaffinized with xylene and hydrated with ethanol at a gradient concentration; after antigen retrieval, cells were blocked with blocking solution for 30 min at room temperature with the primary antibody (Claudin-5, Occludin, ZO-1 diluted 1:200, Abcam, Cambridge, UK). They were incubated at 4 °C overnight and washed 3 times with PBS solution. The secondary anti-goat anti-rabbit IgG antibody (1:1000, BE0101, Bioeasy Biotechnology Inc., Shenzhen, China) was added and the anti-fluorescence attenuation mountant containing DAPI was added dropwise for mounting. The sections were observed under a laser confocal microscope (Zeiss, Oberkochen, Germany) and the field of view was randomly selected for photography.

### 4.15. Immunohistochemistry Assay

Paraffin sections of brain tissue were deparaffinized with xylene, hydrated with ethanol at gradient concentrations, blocked by endogenous enzymes, and blocked by serum. AQP4, GFAP, and BDNF antibodies (diluted at a ratio of 1:500, Abcam, Cambridge, UK) were added dropwise in a 4 °C freezer overnight and, after washing, secondary antibody goat anti-rabbit lgG polymer (1:1000, BE0101, Bioeasy Biotechnology Inc.) was incubated at room temperature for 1 h, DAB was added for color development, and gradient ethanol dehydration, xylene permeabilization, and neutral gum were used to mount the sections. Each slice was photographed and recorded with a random field of view.

### 4.16. Electron Microscopy

Primary ASs and brain tissue were collected, and 1.5 mL of fetal bovine serum was added. After centrifugation at 3000× *g* for 3 min, the supernatant was discarded. One milliliter of 2.5% glutaraldehyde was added, and the cell clumps were fixed for 1 h. The cells were then fixed for 1 h. The cells were washed with PBS solution. The cells were rinsed 3 times using PBS solution for 15 min each time. Next, 0.5 mL of 1% osmic acid (GP18456, Zhongjingkeyi Technology Co., Ltd., Beijing, China) was added and fixed for 1 h. The cells were rinsed 3 times using PBS solution for 15 min each time. Gradient dehydration was performed using different concentrations of acetone, followed by elution with pure acetone solution 3 times for 30 min each. The specimens were next permeabilized using different ratios of anhydrous acetone mixed with embedding agent. The specimens were placed on an embedding plate and polymerized at 45 °C for 12 h, and then, at 60 °C for 48 h. The specimens were then polymerized at 60 °C for 48 h. After embedding, the specimens were cut into with a thickness of 70 nm. Lead citrate (GA1070, Zhongjingkeyi Technology Co., Ltd., Beijing, China) staining was first used for 10 min. Then, the sections were washed with distilled water 3 times for 15 min each time. The sections were then stained with uranium acetate (GZ02625, Zhongjingkeyi Technology Co., Ltd., Beijing, China) for 30 min, followed by 3 washes with distilled water. After the sections were dried, they were observed by transmission electron microscopy and photographed.

### 4.17. Western Blotting

Primary ASs and brain tissue were collected. After extraction of total protein, centrifugation was performed at 12,000× *g* at 4 °C for 5 min, and the supernatant was transferred to a precooled EP tube. Protein quantification was performed using the BCA kit (P0010; Beyotime Institute of Biotechnology). According to the molecular weight of the protein to be tested, 5× loading buffer was added to the protein samples to denature at 95 °C for 10 min. SDS–PAGE gels (Criterion™; Trans-Blot^®^; Bio-Rad Laboratories, Ltd., Hercules, CA, USA) were prepared, and 20 µg of protein sample was added to the gel wells. The electrophoresis was terminated by 80 V stabilized electrophoresis for approximately 30 min to bring the dye to the proper position of the separating gel. A methanol-activated PVDF membrane (Bio-Rad Laboratories, Ltd.) was used as the solid-phase support, and a current of 180 Ma and voltage of 90 V were set to transfer the membrane for 60 min. The membrane was transferred to the closure solution and shaken for 1 h at room temperature. The membrane was placed into the diluted primary antibody working solution, NR1 antibody (1:5000, AB109182, Abcam, Cambridge, UK), NR2C (1:1000, AB192831, Abcam, Cambridge, UK), BDNF (1:1000, BS6533, Bioworld Technology, Inc., Bloomington, MN, USA), CREB (1:3000, AB32515, Abcam, Cambridge, UK), P-CREB (1:3000, ab32096, Abcam, Cambridge, UK), actin antibody (1:10,000, AB0035, ABWAYS Technology, Inc., Shanghai, China), at 4 °C overnight. The membrane was washed 3 times using 1× TBST. The membrane was placed in diluted secondary antibody working solution: goat anti-rabbit IgG H&L (HRP) (1:10,000, BE0101, Bioeasy Biotechnology Inc.) and shaken for 1 h at room temperature away from light. The membrane was washed 3 times with TBST (0.1% Tween, QN1236, Biolab Technology Co., Ltd., Beijing, China). The membranes were fully immersed in ECL chemiluminescent reagent (A38555; Thermo Fisher Scientific, Inc.) for 5 min, and then, exposed with a Tanon 4600 emission chemiluminescent imager. The results were analyzed by using the ImageJ software 1.53t to assess the grayscale values of the bands, and the relative protein expression was defined as the grayscale value of target protein/loading control protein.

### 4.18. Statistics

Data are presented as the mean ± standard deviation (SD) of three repeats. Multiple comparisons of normally distributed data were performed using ANOVA tests, followed by Bonferroni correction. Multiple comparisons with unequal variance were performed using Welch’s ANOVA test followed by Dunnett’s T3 post hoc test. Multiple comparisons of abnormally distributed data were performed using the Kruskal–Wallis test followed by Dunn’s post hoc test. All statistical analyses were performed using the GraphPad Prism 9.0 software, with *p* < 0.05 considered statistically significant.

## 5. Conclusions

In summary, the ischemic stroke model was replicated in vitro and in vivo, and metabolomics analysis technology was used to combine multiple indicators. Based on these results, we conclude that 4HBA can modulate the NMDA-CREB-BDNF pathway by decreasing the metabolite cycloserine, and it can protect ASs and their mitochondria by reducing oxidative stress and restoring the expression of connexin. This facilitates the maintenance of environmental homeostasis in the ischemic region. As an important ingredient in *Gastrodia elata* Bl., gastrodin has been administered intravenously and orally via gel and tablets, but studies have shown that it is difficult for gastrodin to cross the blood–brain barrier to reach the lesion. However, it is rapidly metabolized into 4HBA, with the ability to cross the blood–brain barrier after entering the body. More bioavailable and brain-targeted clinical applications of 4HBA may be developed, and our study will provide more evidence for these. In this study, we focused on ASs, but the protective effect between these and neurons is not well understood. In future work, we will clarify the specific mechanism of 4HBA in the protection of neurons by ASs through co-culture and other methods.

## Figures and Tables

**Table 1:** Screening results of differential metabolites.

Compound	VIP	OGD/R vs. Control		4HBA vs. OGD/R		*p*-Value	KEGG
		FC	Change	FC	Change		
Cycloserine	1.346	44.196	up	0.146	down	0.005	C08057
Betaine aldehyde	1.413	5.5120	up	1.100	up	0.001	C00576
D-Lactic acid	1.813	5.083	up	1.402	up	0.000	C00256
Beta-Sitosterol	1.198	1.994	up	0.564	down	0.019	C01753
Cyclic AMP	1.683	1.837	up	2.863	up	0.022	C00575
L-Phenylalanine	1.441	1.722	up	1.405	up	0.021	C00079
4-hydroxy-2H-chromen-2-one	1.231	1.648	up	1.165	up	0.031	C20414
Xanthurenic acid	1.512	1.313	up	1.262	up	0.031	C02470
(3S,4S)-3-hydroxytetradecane-1,3,4-tricarboxylic acid	1.394	1.268	up	0.780	down	0.013	C04529
Phosphocreatine	1.841	1.070	up	1.355	up	0.012	C02305
D-Ribulose 5-phosphate	1.938	1.068	up	0.765	down	0.005	C00199
2′-Deoxyinosine triphosphate	1.608	1.064	up	1.531	up	0.039	C01345
Azelaic acid	1.844	1.015	up	0.771	down	0.008	C08261
Aniline	1.839	1.013	up	0.580	down	0.011	C00292
16-Hydroxy hexadecanoic acid	1.835	1.010	up	1.793	up	0.013	C18218
Gamma-Tocopherol	1.651	1.000	down	0.787	down	0.029	C02483
Oxidized glutathione	1.737	0.962	down	1.317	up	0.031	C00127
Glutathione	1.971	0.927	down	1.497	up	0.006	C00051
Uridine diphosphate glucose	1.675	0.907	down	1.438	up	0.042	C00029
Hexadecanedioic acid	1.376	0.905	down	0.899	down	0.035	C19615
Cysteinylglycine	2.091	0.872	down	1.645	up	0.002	C01419
L-Glutamine	1.377	0.508	down	1.839	up	0.006	C00064
Spermine	1.309	0.507	down	1.503	up	0.000	C00750
5′-Methylthioadenosine	1.252	0.496	down	1.464	up	0.000	C00170
Cyclic AMP	1.986	0.494	down	2.584	up	0.000	C00575
Citicoline	1.198	0.488	down	1.377	up	0.001	C00307
Succinic acid	1.163	0.486	down	1.531	up	0.004	C00042
Uridine 5′-monophosphate	1.321	0.483	down	1.643	up	0.000	C00105
L-Tyrosine	1.30	0.471	down	1.387	up	0.000	C00082
L-Leucine	1.292	0.466	down	1.480	up	0.000	C00123
Albanin G	1.341	0.453	down	1.781	up	0.001	C10100
Creatinine	1.242	0.437	down	1.400	up	0.000	C00791
NADH	1.244	0.434	down	2.133	up	0.026	C00004
ADP	1.493	0.431	down	2.078	up	0.001	C00008
Spermidine	1.455	0.423	down	2.030	up	0.000	C00315
S-Adenosylhomocysteine	1.047	0.423	down	1.269	up	0.010	C00021
N-Acetylglutamic acid	1.301	0.380	down	1.152	up	0.000	C00624
Biotin	1.299	0.319	down	2.100	up	0.000	C00120
2′-Deoxyguanosine 5′-monophosphate	1.368	0.303	down	2.427	up	0.000	C00362
N-Acetylputrescine	1.558	0.300	down	1.231	up	0.000	C02714
Cytidine	1.099	0.282	down	2.052	up	0.004	C00475
Cycloserine	1.346	44.196	up	0.146	down	0.005	C08057
Betaine aldehyde	1.413	5.5120	up	1.100	up	0.001	C00576
D-Lactic acid	1.813	5.083	up	1.402	up	0.000	C00256
Beta-Sitosterol	1.198	1.994	up	0.564	down	0.019	C01753
Cyclic AMP	1.683	1.837	up	2.863	up	0.022	C00575
L-Phenylalanine	1.441	1.722	up	1.405	up	0.021	C00079
4-hydroxy-2H-chromen-2-one	1.231	1.648	up	1.165	up	0.031	C20414
Xanthurenic acid	1.512	1.313	up	1.262	up	0.031	C02470
(3S,4S)-3-hydroxytetradecane-1,3,4-tricarboxylic acid	1.394	1.268	up	0.780	down	0.013	C04529
Phosphocreatine	1.841	1.070	up	1.355	up	0.012	C02305
D-Ribulose 5-phosphate	1.938	1.068	up	0.765	down	0.005	C00199
2′-Deoxyinosine triphosphate	1.608	1.064	up	1.531	up	0.039	C01345
Azelaic acid	1.844	1.015	up	0.771	down	0.008	C08261
Aniline	1.839	1.013	up	0.580	down	0.011	C00292
16-Hydroxy hexadecanoic acid	1.835	1.010	up	1.793	up	0.013	C18218
Gamma-Tocopherol	1.651	1.000	down	0.787	down	0.029	C02483
Oxidized glutathione	1.737	0.962	down	1.317	up	0.031	C00127
Glutathione	1.971	0.927	down	1.497	up	0.006	C00051
Uridine diphosphate glucose	1.675	0.907	down	1.438	up	0.042	C00029

## Data Availability

The raw data supporting the conclusions of this article will be made available by the authors on request. The metabolomics data presented in the study are openly available in Metabolights at accession number MTBLS9387.
